# Skeletal muscle function underpins muscle spindle abundance

**DOI:** 10.1098/rspb.2022.0622

**Published:** 2022-06-08

**Authors:** Roger W. P. Kissane, James P. Charles, Robert W. Banks, Karl T. Bates

**Affiliations:** ^1^ Department of Musculoskeletal Biology, Institute of Aging and Chronic Disease, University of Liverpool, The William Henry Duncan Building, 6 West Derby Street, Liverpool L7 8TX, UK; ^2^ Department of Biosciences, University of Durham, South Road, Durham DH1 3LE, UK

**Keywords:** muscle spindle, proprioception, biomechanics, MRI, physics simulation

## Abstract

Muscle spindle abundance is highly variable within and across species, but we currently lack any clear picture of the mechanistic causes or consequences of this variation. Previous use of spindle abundance as a correlate for muscle function implies a mechanical underpinning to this variation, but these ideas have not been tested. Herein, we use integrated medical imaging and subject-specific musculoskeletal models to investigate the relationship between spindle abundance, muscle architecture and *in vivo* muscle behaviour in the human locomotor system. These analyses indicate that muscle spindle number is tightly correlated with muscle fascicle length, absolute fascicle length change, velocity of fibre lengthening and active muscle forces during walking. Novel correlations between functional indices and spindle abundance are also recovered, where muscles with a high abundance predominantly function as springs, compared to those with a lower abundance mostly functioning as brakes during walking. These data demonstrate that muscle fibre length, lengthening velocity and fibre force are key physiological signals to the central nervous system and its modulation of locomotion, and that muscle spindle abundance may be tightly correlated to how a muscle generates work. These insights may be combined with neuromechanics and robotic studies of motor control to help further tease apart the functional drivers of muscle spindle composition.

## 1. Introduction

Skeletal muscle is functionally diverse, simultaneously operating as a motor to drive locomotion while also as a sensory organ to detect limb positions and modulate posture [1]. Activation of extrafusal muscle fibres and changes in fascial length enable muscles to produce power. The coordination of activation and fibre length change across multiple muscle groups is a complex process, where the successful execution of a motor command relies on constant proprioceptive feedback to modulate central drivers of movement [1,2]. These proprioceptive signals are detected through two peripherally located sensory apparatuses: muscle spindles and Golgi tendon organs (GTOs) [3]. Muscles differ considerably in their number of muscle spindles and GTOs, however, it is unknown what physiological or functional signal determines this variation.

Measures of the number and distribution of these sensory organs have almost always been derived from muscle histological preparations [4–6], with comprehensive libraries of muscle spindle densities (number of spindles per gram of muscle) in humans having been compiled [7]. By contrast, corresponding data for GTOs have been less well catalogued [8]. Early studies suggested that, in general, smaller muscles contain greater densities of muscle spindles than larger muscles [9]. The higher densities in smaller muscles were often seen as a functional correlate of those muscles being particularly important for fine motor control [6] or functioning as kinesiological monitors [10]. There are however two significant issues: first, these hypotheses were proposed without the support of quantitative functional data, and with subjective descriptions of ‘fine motor control’. Second, the use of muscle spindle density has since been statistically discredited as a meaningful measure to compare muscle spindle composition [11,12]. This negates much of the previously hypothesized underpinning of muscle spindle composition and leaves a substantial gap in our understanding of the functional determinants of muscle spindle composition.

It is now widely established that the relative abundance of muscle spindles is best described using residual values of the linear regression of the log-transforms of spindle number against muscle mass [11,12]. Grouping muscles by anatomical location (e.g. head/neck versus leg/foot) has shown there to be region-specific differences in spindle abundance, with post-cranial and neck muscles containing a significantly greater abundance than those in the legs [11,12]. Additionally, within a single group (e.g. leg) there also exists a diverse distribution of muscle spindle abundances [11]. It is speculated that the motor control strategies employed between and within muscle groups are likely to differ [11,12]. However, to the best of our knowledge, both the mechanistic cause and functional implications of this variation in spindle abundance are unknown. This is in part due to the difficulty in collecting accompanying anatomical data in humans, as samples are largely derived from cadaveric preparations, and muscles being transversely sectioned limits additional morphometric measures that might be used to understand the causes and consequences of variations in spindle abundance. However, medical imaging and computational biomechanical models [13] provide a platform upon which to measure a of variety of anatomical metrics, and to predict functional musculoskeletal metrics that are impossible to measure *in vivo*. Together, these techniques therefore offer the opportunity to investigate the relationships between spindle abundance and other aspects of muscle anatomy (i.e. muscle architecture) and *in vivo* muscle function (i.e. force and power production).

**Figure 1.**
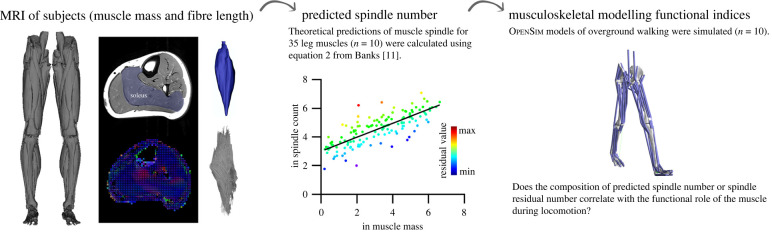
Experimental workflow. 10 participants were MRI scanned to calculate muscle mass and measure median muscle fibre length. Using Banks' [11] equation to predict muscle spindle number, we predicted spindle number (*S*_pn_) for 35 muscles across the lower leg muscles. Subsequently, subject-specific models and simulations were generated for ten participants walking overground, and we conducted correlative analysis of spindle composition with muscle-specific functional indices. (Online version in colour.)

Herein we integrate medical imaging of muscle architecture with subject-specific biomechanical models and simulations to provide the first quantitative tests of correlations between muscle spindle abundance, muscle anatomy and *in vivo* muscle dynamics (figure 1). By combining these outputs with estimates of muscle spindle composition [11,12] we are subsequently able to quantitatively explore the relationship between muscle structure/function and muscle spindle composition for the first time. Specifically, we test if muscle spindle composition correlates with predicted muscle function based on our subject-specific muscle architecture data. In line with previous ideas [6,10,14], it is hypothesized (hypothesis 1) that displacement specialist muscles (composed of long extrafusal fibres and a small physiological cross-sectional area (PCSA)) would contain a greater composition of muscle spindles than those optimized to generate force (short muscle fibres and large PCSA).

Recent *in situ* and mathematical modelling studies have shown primary spindle firing rates to be better predicted by measures of force and yank [1,15,16] when compared to classical measures of muscle length and velocity in a passive muscle. Therefore, to understand if any of these key sensory encoding parameters are predictive of whole muscle spindle composition we performed correlations with indices of gross functional anatomy (i.e. extrafusal fibre length and pennation angle) and dynamic functional data (i.e. muscle length change, velocity of lengthening and force production), all of which are thought to be integral for proprioception [1,11,12,15,17–19]. We therefore hypothesize (hypothesis 2) that muscle spindle composition will not only correlate with measures of fibre length (i.e. anatomical length, length change and velocity of lengthening) but also with the active and passive force kinetics of a muscle.

Finally, to better characterize the relationship between muscle spindle composition and muscle function we have predicted the mechanical work done by the fibres of each individual muscle across one gait cycle, which allowed quantitative descriptors of fibre functional index [20,21] to be correlated with muscle spindle composition. Muscles can be described as either a strut (high force generation but low amounts of work), spring (equal amounts of positive and negative work), motor (high amounts of positive work) or brake (high amounts of negative work). The muscles that make up the triceps surae have some of the lowest abundance of spindles in the lower limb of humans [11] and are thought to predominantly function as struts during walking [21]. It might therefore be hypothesized (hypothesis 3) that muscles with fewer muscle spindles would function primarily as struts, where they generate substantial forces with minimal length change. Conversely, muscles with the greatest composition of muscle spindles may primarily function like brakes, as muscle lengthening is integral to absorb energy [21]. Together, this holistic overview of anatomical and functional correlates with muscle spindle composition provides the most comprehensive analysis of the mechanisms underpinning relative muscle spindle abundance to date.

## 2. Methods

### (a) Published sources

The relationship between muscle mass and spindle number was taken from [11] and used to estimate spindle number (*S*_pn_) using individual muscle mass in this study. Additionally, relative muscle abundance has been taken from Banks' [11] appendix to rank muscles in an objective comparative way. While the relative abundance for each muscle is in some instances derived from low sample numbers (e.g. *n* = 1), there is strong statistical support to show that the relationship between muscle mass and muscle spindle number if drawn from muscles with repeated measures (e.g. *n* > 1) would not significantly deviate from that of the entire catalogue [11] (electronic supplementary material, figure S1a).

Subject data were taken from Charles *et al*. [13], where 10 subjects were recruited (5 male and 5 female; age: 29 ± 3 years; body mass: 67.9 ± 9 kg; height- 175 ± 7 cm; BMI- 21.9 ± 1.6 kg m^−2^) and provided informed consent prior to participating in the study, in accordance with ethical approval from the University of Liverpool's Central University Research Ethics Committee for Physical Interventions (Reference no.: 3757). The muscle spindle data taken from Banks [11] are derived from a broad range in ages (fetal samples – elder specimens) and while our subject range are relatively homogeneous in age and mass it is generally accepted that absolute muscle spindle number does not change throughout ageing [22,23]. The validity of our subject data and subsequent interpretations are supported by the significantly correlated muscle mass against those from Banks [11] (electronic supplementary material, figure S1b; *R*^2^ = 0.97, *p* < 0.001).

### (b) Muscle architecture data

Subject-specific muscle architecture data from 35 muscles of the right lower limb was collected from each subject, as previously described [13]. Briefly, this involves two MRI sequences: a T1-weighted anatomical turbo spin echo (TSE) to estimate muscle volumes and visualize muscle attachment points, and a diffusion tensor imaging (DTI) sequence to estimate muscle fibre lengths and pennation angles. The validity and accuracy of this and similar three-dimensional techniques have been previously established and described elsewhere [13,24].

For each muscle of the lower limb, these median muscle fibre length values from DTI were used to estimate optimal fibre length (*L*_f_) through the normalization to a generic optimal sarcomere length [2.7 µm] [25]. See Charles *et al*. [13] for more details regarding this normalization. These values were then used to calculate PCSA using the following formula:

2.1 $$\mathrm{PCSA}=\;\frac{\left(V_{m}\;\times \cos\theta \right)}{L_{f}},$$

where *V*_m_ is muscle volume from three-dimensional volumetric meshes of each muscle from T1 MR images and *θ* is mean pennation angle.

Muscle mass taken for each individual muscle from the MRI were input into Banks' [11] equation to predict muscle spindle numbers (*S*_pn_) taking into account muscle relative spindle abundance:

2.2 $$S_{\mathrm{pn}}=\;\mathrm{spindle}\;\mathrm{abundance}\;\times \;20.5m_{n}^{0.49},$$

Predicted spindle numbers are plotted for all 10 individuals (electronic supplementary material, figure S1c) overlaid with values taken from Banks [11].

### (c) Subject specific models

These muscle architecture data informed lower limb musculoskeletal models actuated by 92 musculotendon units (MTUs), which were created for each of the 10 participants using nmsBuilder software [26] and subsequently exported to OpenSim 4.1 [27] (figure 1). For more details regarding the creation of these models from MRI, see [13].

Kinematic and kinetic data for walking at self-selected speeds from each individual (mean walking speed = 1.4 m s^−1^) were collected using a 12-camera motion capture system (Qualisys Inc., Göteborg, Sweden) and embedded force plates (Kistler, Winterthur, Switzerland). Within OpenSim, Computed Muscle Control (CMC) [28] was used to predict the lengths, forces, powers and velocities of each MTU, as well as the fibres within each MTU, throughout one walking gait cycle. Furthermore, both the active and passive components of the force exerted by the MTU fibres were predicted separately. In these simulations, residual and reserve actuators were appended to each unlocked degree of freedom to compensate for potential deficiencies in the muscle force-generating properties, as per standard practice [29].

The positive and negative mechanical work generated by the fibres and whole MTU were calculated by integrating the positive and negative portions of the power curves. As described in previous studies [20,21], it is possible to quantify the functional roles of the fibres of each MTU during walking from these work values, using the calculation of four dimensionless functional indices: strut (high force generation but low amounts of work), spring (equal amounts of positive and negative work), motor (high amounts of positive work) and brake (high amounts of negative work), the cumulative percentage of which totalled 100%. Therefore, the functional index with the largest percentage could be considered the primary functional role of the fibres of a particular MTU actuator during the given movement.

The strut indices (*I*_strut_) from the fibres of each MTU in each model condition were calculated as follows:

2.3 $$\begin{array}{r} I_{\mathrm{strut}}=\max\left(1-\;\frac{(t_{\mathrm{FS}}(n+1)-\;t_{\mathrm{FS}}(n))\int_{t_{\mathrm{FS}}(n)}^{t_{\mathrm{FS}}(n+1)}|P_{f}|\;dt}{l_{\mathrm{cha}}\int_{t_{\mathrm{FS}}(n)}^{t_{\mathrm{FS}}(n+1)}|F_{f}|\;dt},\;0\right)\times 100\text{\%}, \end{array}$$

where *t*_FS_ is the time of foot strike, *P*_f_ and *F*_f_ are fibre power and force, respectively, and *l*_cha_ is a characteristic length change factor. The formula of *l*_cha_ is described in detail by [21], but in short it was optimized for each MTU to maximize its spring index relative to its tendon slack length.

A spring-like function of muscle fibres would involve energy absorption, or negative work, during fibre shortening and energy return, or positive work, during lengthening. Therefore, spring indices (*I*_spring_) were calculated as follows:

2.4 $$I_{\mathrm{spring}}=\;\frac{2\times \min(\left|W_{l}^{-}|,\;|W_{s}^{+}\right|)}{\left|W_{\mathrm{tot}}^{-}|+|W_{\mathrm{tot}}^{+}\right|}\;\times \;100\text{\%}-\;I_{\mathrm{strut}},$$

where $W_{l}^{-}$ is the negative work when the fibres are lengthening, $W_{s}^{+}$ is the total positive work when the fibres are shortening, $W_{\mathrm{tot}}^{-}$ is the total negative work and $W_{\mathrm{tot}}^{+}$ is the total positive work. Motor indices (*I*_motor_) were calculated as follows:

2.5 $$I_{\mathrm{motor}}=\;\frac{|W_{\mathrm{tot}}^{+}|-\min(\left|W_{l}^{-}|,|W_{s}^{+}\right|)\;}{\left|W_{\mathrm{tot}}^{-}|+|W_{\mathrm{tot}}^{+}\right|}\;\times \;100\text{\%}-\;I_{\mathrm{strut}}.$$

Brake indices (*I*_brake_) were calculated as follows:

2.6 $$I_{\mathrm{brake}}=\;\frac{|W_{\mathrm{tot}}^{-}|-\min(\left|W_{l}^{-}|,|W_{s}^{+}\right|)\;}{\left|W_{\mathrm{tot}}^{-}|+|W_{\mathrm{tot}}^{+}\right|}\;\times \;100\text{\%}-\;I_{\mathrm{strut}}.$$

### (d) Muscle morphospace plots

We used the morphospace scatterplots to present the relationship between PCSA and *L*_f_ [30–33] and test to see if muscle spindle composition was predictive of muscles functioning as displacement specialists (hypothesis 1). Here, muscles with long and low PCSA (high *L*_f_: PCSA) were classed as ‘displacement specialized’, short *L*_f_ and high PCSA (low *L*_f_: PCSA) as ‘force specialized’ and long *L*_f_ and high PCSA as ‘power specialized’.

### (e) Statistics

Given the recent evidence that muscle force/yank is a better predictor of proprioceptive signalling than absolute fibre length and the rate of length change [1,15], we expect there to be a strong relationship between muscle spindle composition and these physiological variables (hypothesis 2). Muscle spindle composition was correlated with gross anatomical data in an attempt to delineate whether purely structural relationships exist. The length change that muscle spindles will experience depends on several morphometric properties of muscle, namely extrafusal fibre length and fibre pennation [11,18]. Therefore, linear regressions were conducted against estimates of spindle number (*S*_pn_), muscle fibre length and fibre pennation. Next, functionally representative data were correlated to muscle spindle composition, with predictions of fibre strain amplitude, fibre lengthening velocity, active and passive fibre force generated from the biomechanical simulations for each individual muscle. Finally, using the dimensionless functional index, we test to examine if muscle spindle composition is linked with the gross functional capacity of the muscle fibres, where muscles composed of greater spindle composition may be predominantly active and functioning as brakes during fibre lengthening, while those with fewer muscle spindles may function more isometrically like a strut (hypothesis 3). All linear regressions were completed using SPSS (v. 25).

## 3. Results

### (a) Morphometric correlates of muscle spindle composition

**Figure 2.**
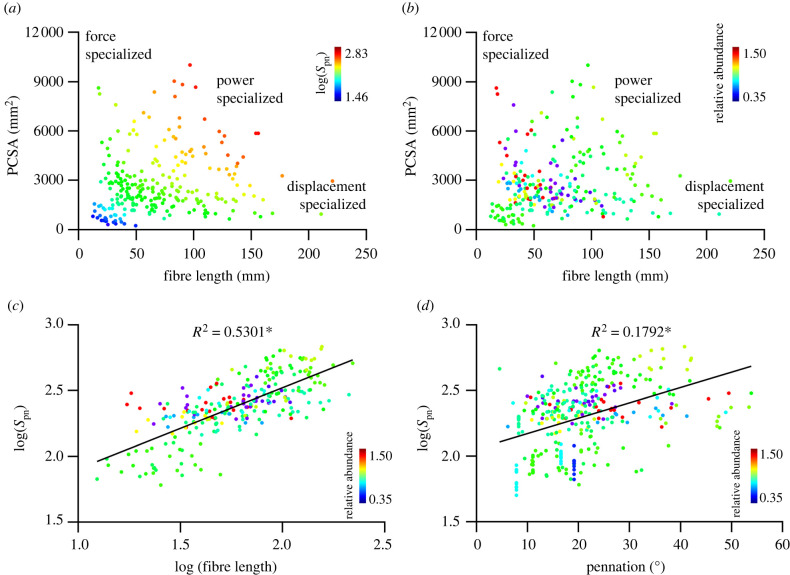
Relationship between spindle composition and morphometric derived specialization. Relationship between muscle fibre length and PCSA across the 10 participants with heat map colouring representing predicted spindle number (*S*_pn_) (*a*) and spindle abundance (*b*). The relationship between log(*S*_pn_) and log(*L*_f_) (*c*) and muscle pennation angle (*d*). *p < 0.05. (Online version in colour.)

Contrary to our first hypothesis, neither absolute muscle spindle number (figure 2*a*) nor relative abundance (figure 2*b*) displayed a localization toward areas of predicted displacement specialist morphospace. Muscles with greater absolute spindle numbers looked to be predominantly optimized to generate power (figure 2*a*), while spindle abundance appeared to present no clear functional division, with a high level of overlap between muscles of greatest and lowest abundance (figure 2*b*). In support of our second hypothesis, absolute muscle spindle number is significantly correlated with both muscle fibre length (*R*^2^ = 0.53, *p* < 0.001; figure 2*c*) and muscle fibre pennation angle (*R*^2^ = 0.18; *p* < 0.001; figure 2*d*). However, there was no clear relationship with muscle spindle abundance (figure 2*c,d*).

### (b) Functional roles and muscle spindle composition

**Figure 3.**
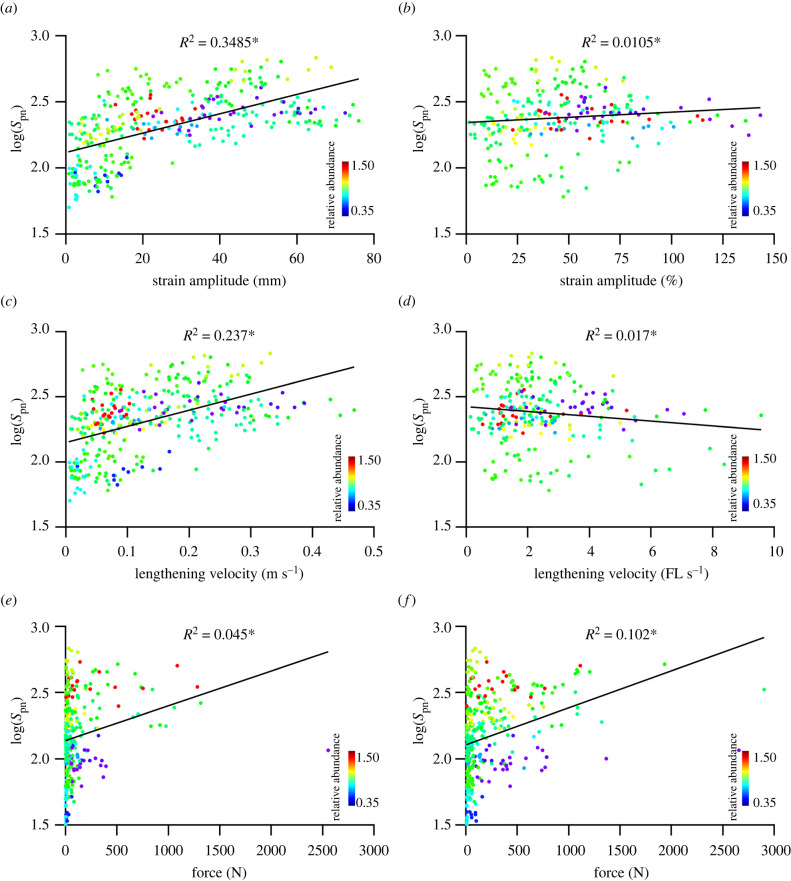
Relationship between spindle composition and modelled muscle length and force characteristics during walking. Predicted spindle counts plotted against absolute strain amplitude (*a*), relative strain amplitude (*b*), maximum absolute lengthening velocity (*c*), maximum normalized lengthening velocity (*d*), maximum passive fibre force (*e*) and maximum active fibre force (*f*). *p < 0.05. (Online version in colour.)

Significant positive relationships exist between muscle spindle number and both the absolute strain amplitude (*R*^2^ = 0.35, *p* < 0.001; figure 3*a*) and the absolute lengthening velocity (*R*^2^ = 0.24, *p* < 0.001; figure 3*c*) of a given muscle, which further supports our second hypothesis. Conversely, normalization of strain amplitude to a percentage of muscle length (*R*^2^ = 0.004, *p* = 0.01; figure 3*b*) ablated any relationship with spindle number, while velocity normalized to fibre lengths maintained a small but significant trend (*R*^2^ = 0.02, *p* < 0.05; figure 3*d*). Despite significant relationships between absolute spindle number and absolute strain amplitude and velocity, there does not appear to be any correlation with the abundance of muscle spindles (figure 3). Moreover, passive (*R*^2^ = 0.045, *p* < 0.001; figure 3*e*) and active (*R*^2^ = 0.102, *p* < 0.001; figure 3*f*) fibre forces experienced by individual muscle fibres appeared to present with a weak but significant positive correlation with muscle spindle number, while no such trend existed with muscle spindle abundance (figure 3*e,f*).

**Figure 4.**
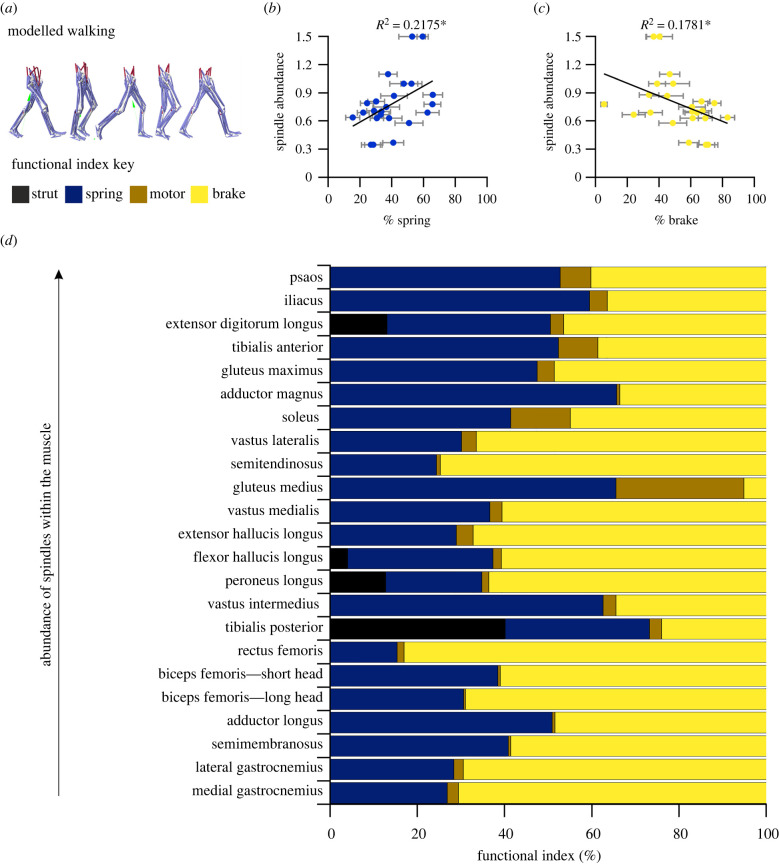
Relationship between spindle composition and functional indices during walking. Representative OpenSim modelled gait cycle (*a*). Relationship between muscle spindle abundance and the percentage of functional indexes for spring (*b*) and brake (*c*) behaviour. The average functional index for individual muscles from the 10 participants ranked in order of spindle abundance (*d*). *p < 0.05. (Online version in colour.)

Looking at the gross *in vivo* function of muscle fibres throughout an entire gait cycle (figure 4*a*) we see that quantitative descriptors of muscle function may be linked to muscle spindle abundance (figure 4). Functional indices varied across individual muscle fibres (electronic supplementary material, figures S1 and S2) with the adductor magnus and gluteus medius predominantly functioning as springs throughout walking (spring indices of 65.7 ± 6.2% and 65.4 ± 5.2%, respectively), compared to the rectus femoris and medial gastrocnemius functioning predominantly as brakes (brake indices of 83.1 ± 4.3% and 70.7 ± 6.2%, respectively). Counter to expectations (hypothesis 3), across all muscle fibres studied in each subject, there was a significant positive relationship between spring-like function and the abundance of muscle spindles (*R*^2^ = 0.22; *p* = 0.025, figure 4*b,d*), and a more moderate but significant inverse relationship between muscles that act with a brake-type function and spindle abundance (*R*^2^ = 0.18, *p* = 0.045; figure 4*c,d*). This relationship held true when looking at the function index of the entire MTU (electronic supplementary material, figure S3) with muscle spindle abundance exhibiting a positive significant relationship to spring-like function (*R*^2^ = 0.1934, *p* = 0.036; electronic supplementary material, figure S3b) and brake like function negatively correlating with spindle abundance (*R*^2^ = 0.1385, *p* = 0.08; electronic supplementary material, figure S3c).

## 4. Discussion

There exists an inherent difficulty in the exploration of correlates of muscle spindle abundance and motor function, especially across human subjects where data are exclusively derived from serial muscle histology preparations [11,14,34]. This methodological approach limits the number of morphometric indices available and any potential to correlate muscle spindle abundance with muscle functional outputs. However, here we have uniquely incorporated the datasets accrued over the past century with a subject-specific medical imaging and modelling approach to unpick the potential physiological mechanisms underpinning muscle spindle abundance, which could ultimately inform research into neuromechanics, robotics and clinical interventions for various muscular dystrophies [35–37]. Here we show that contrary to expectations [6,10,34], muscles composed of a greater number of spindles are not anatomically aligned to those specialized for displacement, while the absolute number of muscle spindles is significantly correlated with muscle fibre length and pennation angle. In addition, we demonstrate for the first time that muscle spindle abundance may be associated with the functional role of the skeletal muscle, with fibres that predominantly function as springs exhibiting a greater abundance of spindles relative to muscles whose fibres' primary role is to act as brakes.

### (a) Morphometric determinants of muscle spindle composition

In an attempt to identify trends between muscle architecture, absolute spindle number and relative spindle abundance we used heat mapped morphospace scatterplots (figure 2) to differentiate predicted functional specializations of muscles [30–33]. While a subtle trend exists for muscles with greater absolute numbers of spindles to be optimized for generating power, there was a lack of any definitive clustering when looking at spindle abundance (figure 2). This refutes suggestions that absolute spindle number correlates with muscles optimized for displacement function and sensing (hypothesis 1). However, our work here does provide the first statistical support of previously suggested relationships between muscle spindle number and muscle fibre length and pennation [11,18,19]. Muscles with greater fascicle lengths appear to contain greater numbers of muscle spindles, as do muscles with a greater degree of pennation (figure 2*c,d*). This suggests that muscle spindle number may be tightly regulated to the length of fascicles, and supports our second hypothesis that fibre length is an essential signal input to the central nervous system [18,38].

### (b) Functional correlates of spindle abundance

Muscle spindle density as an indicator of spindle abundance has often been used to hypothesize the functional roles of muscles [14,34] despite no evidence of or tests for a correlation with muscle function. The data presented here begin to fill this gap in understanding. First, we showed that absolute muscle spindle number positively correlates with both absolute muscle strain amplitude and lengthening velocity during walking (figure 3*a,c*). Additionally, we recovered correlations between muscle force experienced during walking with muscle spindle number (figure 3*e,f*). These data suggest that fibre length change and the velocity with which it changes are important physiological variables for the central nervous system and further support our second hypothesis. Moreover, we demonstrate that the force muscles exert during locomotion may be tightly correlated with the composition of muscle spindles, adding further support towards the importance of force as a proprioceptive signal [1,15]. It might be expected, given our novel correlations of muscle fibre lengths with estimated spindle number, that absolute strain amplitude would also correlate with spindle number. While a positive correlation does exist (electronic supplementary material, figure S4), the wide distribution of spindle number (electronic supplementary material, figure S4a) suggests that this relationship (figure 3) is not entirely explained by fibre length. Additionally, the correlations seen between spindle number and fibre force suggest that the drivers behind absolute spindle number within a muscle are more complex. That said, these data do not differentiate a driving mechanism for the relative abundance of muscle spindles (figures 2 and 3; electronic supplementary material, figure S4b).

Therefore, we explored the utility of a more comprehensive overview of muscle function through the characterization of muscle functional indices [20,21]. This quantitative approach provides a descriptive mechanical function for muscle fibres and the entire MTU, incorporating temporal aspects of force generation and muscle length change that we have now shown to be important predictors of muscle spindle composition. This may begin to unpick the underlying physiological function of muscle spindle abundance. Our analyses suggest that a quantitative relationship exists between the gross *in vivo* function of a muscle and muscle spindle abundance, with muscles containing a high abundance predominantly functioning as springs during overground walking, in contrast to those less abundant in spindles, which typically function more like brakes (figure 4; electronic supplementary material, figure S3). From these data, we reject our third hypothesis and instead infer that a muscle with greater spindle abundance may not only absorb a large amount of energy during lengthening (negative work) but also generate substantial force during shortening (positive work), in comparison to muscles with a lower abundance that would primarily generate force during lengthening (negative work) and produce negligible positive work. These data further support the importance of force as an integral signal to the muscle spindle [1,15,16], with muscles most highly abundant in spindles generating force during both lengthening and shortening, compared to muscles that are less abundant generating force primarily during lengthening. However, it should be acknowledged that the impressive predictability of force and yank is primarily of primary afferent firing, not that of secondary endings—which are often more numerous in the muscle spindle [39]—confounding the importance of force and yank as determinants of spindle abundance.

Given the correlations recovered here, it would be interesting to study a greater range of locomotor behaviours, specifically including external physical perturbations, as muscle functional behaviours and the sensorimotor response are likely to differ during postural disturbance [15,21]. However, this would require considerably more experimental data and more sophisticated modelling approaches to govern control of the model [40,41]. Additionally, the opportunity to implement similar analyses across a greater range of body regions—such as in the head and neck muscles [42,43], which are known to have the greatest abundance of muscle spindles in the human body [11]—would shed further light on the physiological underpinning of muscle spindle abundance. Arguably the greatest limitation of the current study is the use of estimated muscle spindle numbers, though our subject muscle mass significantly correlated with those of Banks [11] (electronic supplementary material, figure S1b) and as such our predicted values show strong overlap with measured values in the literature (electronic supplementary material, figure S1c). To date, it is still impossible to image muscle spindles *in situ* (especially in human subjects), though recent progressions in medical imaging have shown that it is possible to segment muscle spindle capsules and individual intrafusal fibres of the mouse soleus [44]. With further developments and improved resolution of medical imaging techniques, it may one day be possible to differentiate the more viscus-filled spindle capsules from the surrounding extrafusal fibres [4]. However, the recent identification of molecular signatures for each individual afferent subtype (muscle spindle; groups Ia, II and Golgi tendon organs; Ib) [45] may pave the way to better understanding of the organization and function of the proprioceptive system during locomotion in animal models. Finally, our data may provide guidance to better tailor physiotherapy strategies for trauma/pathologically impaired proprioception, where there there is no gold standard exercise rehabilitation protocol to promote recovery; rather a combination of exercise tasks is used to challenge the CNS for rehabilitation [46]. Our data might suggest that the use of passive movements as a rehabilitation exercise is not functionally important and that exercise tasks that involve eccentric (brake-like) movements would be more beneficial to a sub-population of muscles, compared to others that may benefit from more concentric exercises. While highly speculative, it might be that our better understanding of key encoding signals integral to proprioception (at least structurally) could inform the development and optimization of actuators in active prosthetics or legged robots to tune their drive/function more effectively during locomotion, as well as increase stability over uneven or compliant terrains.

## 5. Conclusion

Despite the identification of the muscle spindle over a century ago, we still lack a fundamental understanding of the functional drivers of muscle spindle abundance. In addition, we know even less about the functional underpinning of the GTOs that may share similar structural/functional underpinnings to the muscle spindle. This work highlights the power of integrated medical imaging and musculoskeletal modelling to experimentally explore relationships between physiological form and function. Here we have shown for the first time that the number of muscle spindles is closely coupled to the fibre lengths of a given muscle, and predictive of muscle length, velocity excursions and to some extent the force-generating capacity of a muscle during walking. We highlight that, contrary to previously held beliefs, muscles with greater numbers of spindles are not built to function as displacement specialists. Finally, we present the first evidence of spindle abundance-associated muscle fibre function, whereby muscles more abundantly supplied with muscle spindles work primarily as springs, and those less abundantly supplied typically function as brakes during overground walking. This work suggests potentially new roles for muscle spindles in proprioception and may provide guidance to better tailor physiotherapy strategies for trauma/pathologically impaired proprioception, as well as to design more effective actuators to optimize the stability of legged robots or active prosthetics.

## Data Availability

All raw data are available in electronic supplementary material, data file. Subject-specific models are already publicly available online: http://datacat.liverpool.ac.uk/1105/. The data are provided in electronic supplementary material [47].
